# Randomised clinical studies investigating immediate and short-term efficacy of an occluding toothpaste in providing dentine hypersensitivity relief

**DOI:** 10.1186/s12903-019-0781-x

**Published:** 2019-06-04

**Authors:** Jonathan Creeth, John Gallob, Farzana Sufi, Jimmy Qaqish, Paola Gomez-Pereira, Chandrashekhar Budhawant, Chhaju Goyal

**Affiliations:** 1 GSK Consumer Healthcare, Weybridge, Surrey UK; 2 Silverstone Research, Las Vegas, NV USA; 3 All Sum Clinical Research, Mississauga, Ontario Canada; 4 Syneos Health Clinical, Hyderabad, India

**Keywords:** Dentin hypersensitivity, Tin fluorides, Clinical trial, Toothpastes

## Abstract

**Background:**

Dentine hypersensitivity (DH) can occur after gum recession or enamel loss and may impact quality of life. Treatments include toothpastes that decrease DH by occluding dentine tubules. One effective occluding ingredient used in toothpastes is stannous fluoride (SnF_2_), but this can be unstable in aqueous formulation. These three studies aimed to characterise the short-term effects of an experimental, anhydrous SnF_2_ dentifrice on DH.

**Methods:**

Three examiner-blind, parallel-group studies evaluated DH in participants with the condition after a single brushing and after 3d brushing with an experimental anhydrous 0.454% SnF_2_/polyphosphate toothpaste (Test) or a toothpaste containing 0.76% sodium monofluorophosphate (Control). Test treatment participants brushed two pre-identified sensitive teeth first, then their remaining dentition for ≥1 min (‘focused brushing’). Control treatment participants brushed their whole dentition for ≥1 min. DH was measured after single brushing and after 3d twice-daily use, via evaporative (air) (Schiff Sensitivity Scale) and tactile (Yeaple probe) stimuli and analysed using an ANCOVA model.

**Results:**

In all studies, after 3d treatment, the Test toothpaste/brushing regimen significantly reduced DH compared to the Control regimen by both evaporative and tactile stimuli assessment (*p* < 0.0001 for all). The Test regimen also significantly reduced DH from baseline at both time-points by both measures in all studies (p < 0.0001 for all).

Mean Schiff sensitivity score differences (95% confidence intervals) between Test and Control regimens after 3d were: Study 1: − 0.45 (− 0.577, − 0.319); Study 2: − 0.40 (− 0.505, − 0.300); Study 3: − 1.31 (− 1.500, − 1.128). Mean tactile score differences were: Study 1: 11.30 (7.927, 14.662); Study 2: 3.57 (2.531, 4.614); Study 3: 24.54 (20.349, 28.736). After single use, in Studies 2 and 3, the Test toothpaste/brushing regimen significantly reduced DH versus Control by both measures (*p* < 0.001 for all); in Study 1, treatment differences were not significant. Toothpastes were generally well-tolerated.

**Conclusions:**

Taken together, these studies indicated focused brushing with an experimental anhydrous 0.454% SnF_2_/polyphosphate toothpaste reduces DH compared to brushing with a conventional toothpaste after single use, with greater reduction after 3d.

**Trial registration:**

Registrations at ClinicalTrials.gov: Study 1: NCT02832375 (registered 26.July.2016); Study 2: NCT02731833 (registered 26.April.2016); Study 3: NCT02923895 (registered 5.October.2016).

## Background

Gum recession or enamel loss can lead to the underlying dentine becoming exposed [1, 2]. A thermal, tactile, chemical, osmotic or evaporative stimulus, such as a hot drink or cold air, is believed to lead to movement of the fluid contained within dentine tubules, which in turn stimulates pupal nerve fibres and results in a short, sharp pain characteristic of dentine hypersensitivity (DH), as detailed in the ‘hydrodynamic theory’ of DH [3–5]. DH has been shown to have a negative impact on people’s oral health-related quality of life [6], affecting such aspects as eating, drinking, talking and participating in outdoor activities [7].

There are two predominant methods to treat DH using oral care products: nerve desensitisation and tubule occlusion. Potassium ions (a nerve desensitiser) [8, 9] may need repeated administration over a number of weeks before symptomatic relief is achieved [10]. The latter method, occlusion of dentine tubules, relies on ingredients such as strontium or stannous salts [8, 11, 12], arginine-calcium carbonate [9, 13] or bioglasses [14, 15]. These form solid deposits in the exposed ends of dentine tubules that physically block them so an external stimulus does not reach the fluid held within [8–10]. This approach has the potential to work more rapidly than desensitisation.

Previous studies of anti-sensitivity formulations containing occluding agents have found advantages for short-term relief of DH when the toothpaste is applied in a focused manner to the affected sensitive teeth. This may be achieved by massaging a small amount of toothpaste into each sensitive area via a finger-tip (the ‘dab-on’ technique) [12, 16–20] or by ‘focused brushing’ of the affected teeth, where the sensitive areas are brushed with toothpaste before the rest of the dentition [21–24]. The ‘focused brushing’ technique is of interest as it is easily incorporated into a normal toothbrushing routine.

Stannous fluoride (SnF_2_) is a particularly interesting option as an occluding agent as it provides both the potential for sensitivity relief and a source of free fluoride ions for protection against dental caries. It is also compatible with most other conventional toothpaste ingredients and is relatively taste-neutral. The stannous ion is the active component which rapidly oxidises (from Sn[II] to Sn[IV]) and hydrolyses in the presence of saliva to form insoluble tin compounds (hydroxides, oxides and phosphates) [25]. Stannous ions have been shown in vitro to form insoluble precipitates on the dentine surface that occlude dentine tubules through combination with formulation excipients and saliva-derived ions [25, 26].

SnF_2_ has been used in toothpastes to relieve DH for many decades [27] and has shown evidence of clinical efficacy in both short-term and long-term studies [12–24, 27–30]. Since the discovery of the anti-DH properties of SnF_2_, toothpaste formulation development in the industry has concentrated on maximising stability (avoiding premature oxidation and hydrolysis) and delivery to the site of action, while minimising the staining effects of stannous ions [31, 32]. Use of anhydrous formulations of SnF_2_ leads to products that are stable on storage but readily hydrate and become active on exposure to saliva and the aqueous oral environment [33–35]. The addition of polyphosphates can control stannous ion-induced dental stain build-up [36]. This SnF_2_ with polyphosphate anhydrous formulation approach has been shown to occlude dentine tubules in vitro [33, 37, 38], with clinical studies over 8 wk. showing a reduction in DH [24, 27] and limitation of dental stain build-up [36]. However, not all published data for DH relief after just a single focused brushing is positive [29].

Further development of the anhydrous SnF_2_ formulation has since been completed by GSK Consumer Healthcare. This work aimed to improve the ability of the formulation to deliver stannous ions rapidly to the site of action at the dentine surface and retain it there by optimising the polymer thickening system. The resulting new experimental formulation has been shown in vitro to increase the rate of tubule occlusion compared to an existing anhydrous SnF_2_/polyphosphate formulation (unpublished findings).

Following these in vitro results, clinical studies were needed to investigate the effects of the new experimental formulation on DH. Three such studies were undertaken, which are the subject of this report. The aim of these three studies was to evaluate the ability of an experimental 0.454% SnF_2_/sodium tripolyphosphate (STP) anhydrous toothpaste to provide relief from DH when applied by the ‘focused brushing’ technique prior to whole mouth brushing, as elicited by evaporative (air) and tactile stimuli after 3 d twice-daily use and after a single application, compared to a regular toothpaste containing 0.76% sodium monofluorophosphate (SMFP) applied by conventional toothbrushing.

## Methods

These three studies closely followed the protocol described in a previous investigation by some of the current authors (Gallob et al. [39]). The studies used a 3 d, randomised, examiner-blind, two treatment-arm, parallel design. In accordance with consensus guidelines [40], two independent, stimulus-based clinical measures were used to assess DH: evaporative (air) sensitivity (Schiff Sensitivity score [41]) and sensitivity to tactile stimulus (via a constant-pressure Yeaple probe [42]). Study 1 was conducted by Silverstone Research Group, USA; Studies 2 and 3 were conducted by All Sum Research Centre, Canada (on separate populations). All studies were conducted in accordance with the Declaration of Helsinki and approved by independent institutional review boards before initiation (Study 1: US Institutional Review Board, Inc., Miami, FL, USA, Reference number: U.S.IRB2016SRG/01; Study 2: Veritas IRB, Montreal, Canada, Reference number: 16045–16-02:5011-03-2016; Study 3: Veritas IRB, Montreal, Canada Reference number: 16087–12:05:1921-12-2016). These studies are registered at ClinicalTrials.gov: Study 1: NCT02832375; Study 2: NCT02731833; Study 3: NCT02923895.

### Participants

Criteria for eligibility for the studies were as used in the previous investigation [39], modified as described below.

Inclusion criteria: participants needed to be 18–65 yr. with a self-reported history of DH lasting between 6 mth and 10 yr., with at least two accessible teeth (incisors, canines or premolars, non-adjacent) with a positive response to an evaporative air stimulus [41], that had a Modified Gingival Index (MGI) [43] score of 0, signs of facial/cervical erosion, abrasion and/or gingival recession (EAR) and a clinical mobility score ≤ 1 [44].

Exclusion criteria: participants in Study 2 could not participate in Study 3; professional desensitising treatment within 8 wk. of Screening was not a stated exclusion criterion, other than for test teeth in study 3; and certain general dental exclusions in the previous study applied to test teeth only in this case (exposed dentine with deep, defective or facial restorations; teeth used as abutments for fixed/removable partial dentures; full crowns or veneers; orthodontic bands; cracked enamel and sensitive teeth with contributing aetiologies other than EAR; presence of dental implants in Study 3 only).

### Procedures

Procedures at the screening visit followed those of Gallob et al. [39]; dentition was assessed for eligibility as described above.

Eligible participants were supplied with a conventional fluoride toothpaste (Crest Cavity Protection; Procter & Gamble, Cincinnati, OH; USA; USA/Canadian marketed product; 1000 ppm fluoride as sodium fluoride) and a toothbrush (Studies 1 and 2: Aquafresh Clean Control [Everyday Clean], GSK Consumer Healthcare, Weybridge, UK [GSKCH]; Study 3: Oral-B Sensi-soft Manual Toothbrush; Proctor & Gamble, Cincinnati, OH, USA). Following first use under study site supervision, participants used this toothpaste twice daily during the acclimatisation period between screening and baseline visits: 4–6 wk. (Studies 1 and 2), 4–8 wk. (Study 3).

Procedures for the Baseline and 3 d visits also followed those of Gallob et al. [39], other than: participants refrained from taking analgesics for at least 8 h prior to the study visit, and subjects were not permitted to chew gum during the study. In each study, a single examiner performed all assessments for the duration of the trial.

Eligible participants were assigned randomly to one of two toothpaste/brushing regimens: one using the ‘Test’ toothpaste, containing 0.454% SnF_2_ (1100 ppm fluoride) and 5% STP, the other using the ‘Control’ toothpaste, containing 0.76% SMFP (1000 ppm fluoride) (Colgate Cavity Protection; Colgate-Palmolive, New York, NY, USA; USA marketed product).

Subjects were stratified according to their maximum baseline Schiff sensitivity score of the two selected teeth, to ensure balance between the Test and Control regimen groups. Subjects with maximum baseline Schiff sensitivity score of 2 for the two selected test teeth were allocated to Stratum 1; those with maximum score of 3 were allocated to Stratum 2. Randomisation numbers within each stratum were assigned in increasing numerical order, according to appearance at the study site on the day subjects were randomised, once eligibility was determined. Assignment was performed according to a randomisation schedule provided by the Biostatistics department of the study sponsor.

Toothpaste/brushing regimen allocation was kept blind to the dental examiner, study statistician, data management staff and other employees of the sponsor who could have influenced study outcomes. To help blind participants to product identity, study product tubes were overwrapped with white vinyl.

Clinical assessments of DH to evaporative (air) and tactile stimuli were made after the first use of the assigned toothpaste, at the study site. All participants brushed with a full ribbon of toothpaste. In Studies 1 and 2, participants in the Test treatment group were instructed to brush each of the two test teeth first, then the whole dentition for at least 1 min. The duration of focused brushing was not specified in studies 1 and 2; in Study 3 (which occurred after studies 1 and 2), it was set at 30 s per tooth, to standardise product use [21–23]. Control group participants in all studies were instructed to brush the whole dentition for at least 1 min. All participants were permitted to rinse with tap water following brushing.

Participants then brushed twice a day (morning and evening) in this manner for 3 d, following which DH assessments were repeated. Compliance with use of the study toothpaste was assessed by review of participant-completed diary cards.

### Safety

Adverse events (AEs) and any abnormalities in the OST examination were recorded from the start of use with the acclimatisation toothpaste at the screening visit until 5 d after the last use of study toothpaste. The investigator assessed whether an AE was treatment-related or not and graded it as mild, moderate or severe.

### Data analysis

Based on outcomes from previous sensitivity studies (unpublished findings), for Study 1, it was planned to screen sufficient participants to enter approximately 250 into the acclimatisation phase so as to randomise approximately 240 and ensure approximately 214 participants completed the study. For Study 2, participant numbers were: acclimatisation 240; randomisation 235; for 214 to complete. For Study 3, participant numbers were: acclimatisation 210; randomisation 190; for 184 to complete (in which the sample size was also based on the results of Studies 1 and 2, which preceded it, and unpublished findings).

The primary endpoint was change from baseline (mean of the two selected test teeth) in evaporative (air) sensitivity after 3 d, as recorded on the Schiff Sensitivity Scale. Secondary endpoints were: change from baseline in Schiff sensitivity score after a single application; change in tactile threshold after a single application; and change in tactile threshold after 3 d. The primary objective was to determine whether there was a difference in primary endpoint between the Test and Control toothpaste/brushing regimens, the null hypothesis being that there was no difference.

For Studies 1 and 2, it was estimated that using 107 participants per group would have an 80% power to detect a mean difference between treatments of 0.25 units in Schiff sensitivity score (assuming a standard deviation [SD] of 0.6487) using a two-sided t-test of significance level 0.05. This represents a clinically significant difference after 3 d of treatment. For Study 3, it was estimated that a sample of 92 participants per group would have a 90% power to detect a mean difference between the treatments of 0.25 units in Schiff sensitivity score (assuming a SD of 0.5198) using the same significance level.

Efficacy analyses were performed on the intent-to-treat (ITT) population, defined as all randomised participants who provided at least one post-baseline assessment of efficacy. The per-protocol (PP) population was defined as all participants in the ITT population who had at least one efficacy assessment unaffected by protocol violations.

Change from baseline was evaluated by analysis of covariance (ANCOVA), with treatment group as a factor and the corresponding baseline score (Schiff sensitivity score or tactile threshold) as a covariate. For tactile threshold, the maximum baseline Schiff sensitivity score stratum of the two selected test teeth was included as a factor. For all treatment groups, baseline means, adjusted means for the between-treatment differences, 95% confidence intervals (CIs) and *p*-values for treatment comparisons are presented. All tests were conducted at the two-sided 5% significance level, with no adjustments for multiple testing as the primary comparison was pre-defined.

## Results

The ANCOVA model assumptions for the analyses of Schiff sensitivity score were investigated and considered to be satisfied for all studies. For the tactile threshold data, there was some evidence of departure from the model assumptions, therefore change in tactile threshold was also analysed by a non-parametric method (van Elteren test, adjusting for the maximum baseline Schiff sensitivity score) and the results compared with the ANCOVA results. The inferences from the two analyses were similar, thus emphasis has been given to the ANCOVA results.

### *Participants:* Tables 1 and 2.

For Study 1, 242 participants were randomised to treatment. The first participant was enrolled on 28 March 2016, the last completed the study on 12 May 2016. Of the 242 participants in the safety population, the majority were female (*n* = 154; 63.6%) and were in Schiff stratum ‘3’ (*n* = 177; 73.1%); the mean age was 37.7 yr. (SD: 11.15; range 20–65 yr).

**Table 1:** Participant disposition through study

	Number of participants					
	Study 1		Study 2		Study 3	
Enrolment						
Total screened	266		229		197	
Randomised	242		222		192	
Not randomised	24		7		5	
Not eligible	14		7		1	
Withdrew consent	9		0		4	
Lost to follow-up	1		0		0	
Allocation	Test	Control	Test	Control	Test	Control
Randomised/received	121	121	111	111	97	95
Follow-up						
Completed study	119	121	111	111	97	95
Withdrew consent	1	0	0	0	0	0
Lost to follow-up	1	0	0	0	0	0
Analysis						
Safety/ITT/PP populations	121	121	111	111	97	95

**Table 2:** Summary of demographic and baseline characteristics (safety population)

Characteristic	Study 1		Study 2		Study 3	
	Test (*n* = 121)	Control (n = 121)	Test (*n* = 111)	Control (n = 111)	Test (*n* = 97)	Control (*n* = 95)
Sex, n (%)						
Male	45 (37.2)	43 (35.5)	29 (26.1)	30 (27.0)	29 (29.9)	23 (24.2)
Female	76 (62.8)	78 (64.5)	82 (73.9)	81 (73.0)	68 (70.1)	72 (75.8)
Age, yr						
Mean (SD)	38.0 (11.32)	37.4 (11.01)	46.4 (10.77)	47.6 (10.95)	46.8 (12.95)	46.9 (10.53)
Range	20–66	20–64	19–64	18–65	19–65	19–65
Race, n (%)						
White	74 (61.2)	72 (59.5)	60 (54.1)	73 (65.8)	62 (63.9)	51 (53.7)
Black/African American	25 (20.7)	27 (22.3)	33 (29.7)	23 (20.7)	18 (18.6)	31 (32.6)
Asian	9 (7.4)	10 (8.3)	18 (16.2)	15 (13.5)	16 (16.5)	12 (12.6)
Other	13 (10.7)	12 (9.9)	0	0	1 (1.0)	1 (1.1)
Schiff stratum 2, n (%)	33 (27.3)	32 (26.4)	52 (46.8)	51 (45.9)	45 (46.4)	43 (45.3)
Schiff stratum 3, n (%)	88 (72.7)	89 (73.6)	59 (53.2)	60 (54.1)	52 (53.6)	52 (54.7)

For Study 2, 222 participants were randomised to treatment. The first participant was enrolled on 4 April 2016, the last completed the study on 20 May 2016. Of the 222 participants in the safety population, the majority were female (*n* = 163; 73.4%) and were in Schiff stratum ‘3’ (*n* = 119; 53.6%); the mean age was 47.0 yr. (SD: 10.85; range 18–65 yr).

For Study 3, 192 participants were randomised to treatment. The first participant was enrolled on 11 October 2016, the last completed the study on 16 December 2016. Of the 192 participants in the safety population, the majority were female (*n* = 140; 72.9%) and were in Schiff stratum ‘3’ (*n* = 104; 54.2%); the mean age was 46.9 yr. (SD: 11.79; range 19–65 yr).

The Baseline characteristics of the treatment groups were similar between groups for the safety and ITT populations of each study: in none of the studies was there a difference between the ITT and Per-Protocol populations.

### Efficacy

**Fig. 1:**
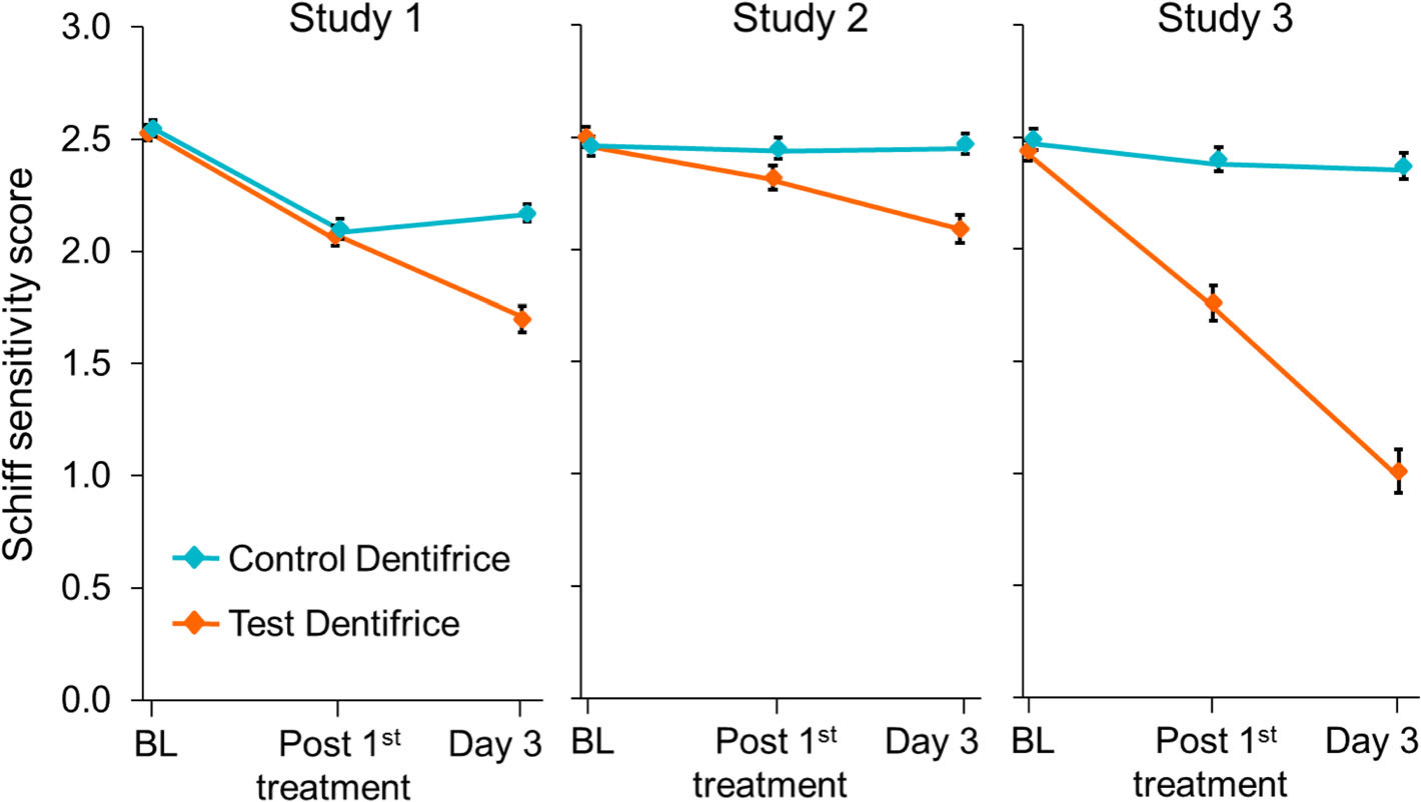
Mean (± standard error) Schiff sensitivity scores (intent-to-treat population). Data are offset for clarity. BL: Baseline

**Fig. 2:**
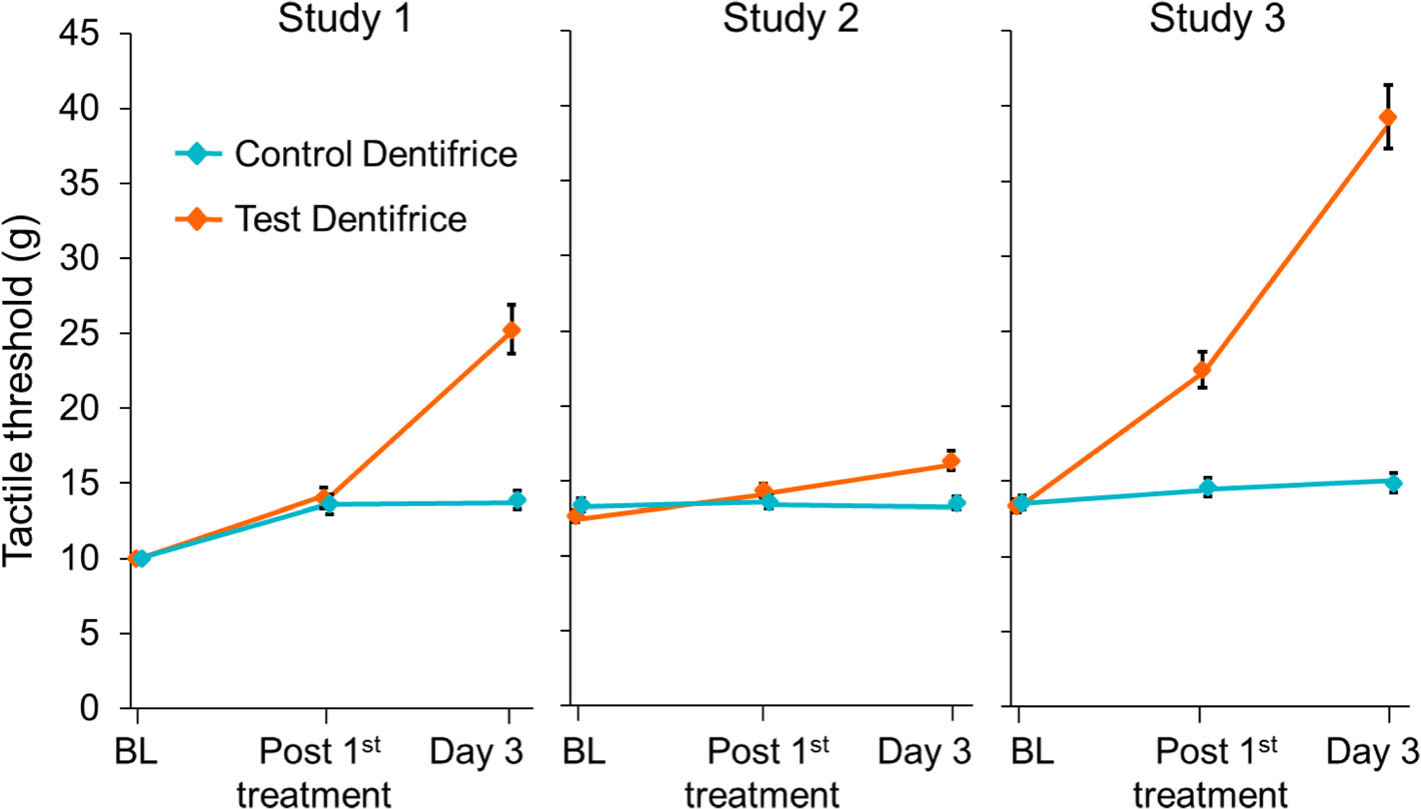
Mean (± standard error) tactile threshold scores (intent-to-treat population)**.** Data are offset for clarity. BL: Baseline

In all studies, the Test toothpaste/brushing regimen showed a statistically significant decrease from baseline in Schiff sensitivity scores (Fig. 1) and increase from baseline in tactile threshold (Fig. 2) after both single and 3 d use (*p* < 0.0001 for all) (Table 3). In Study 1 only, the Control toothpaste/brushing regimen showed a statistically significant reduction in DH from baseline, as detected via both measures at both timepoints (*p* < 0.002 for all).

**Table 3:** Statistical analysis of change from baseline in Schiff sensitivity score and tactile threshold (intent-to-treat population)

		Test*	Control*	Test vs Cont**
Schiff sensitivity score				
Study 1	BL	2.53 (0.035)	2.55 (0.036)	
	0 d	−0.46 (− 0.55, − 0.38) ***p*** **< 0.0001**	−0.45 (− 0.53, − 0.37) ***p*** **< 0.0001**	−0.01 (− 0.131, 0.102) *p* = 0.8067
	3 d	−0.83 (− 0.92, − 0.74) ***p*** **< 0.0001**	−0.38 (− 0.47, − 0.29) ***p*** **< 0.0001**	−0.45 (− 0.577, − 0.319) ***p*** **< 0.0001**
Study 2	BL	2.5 (0.046)	2.46 (0.044)	
	0 d	−0.17 (− 0.23, − 0.11) ***p* < 0.0001**	−0.02 (− 0.08, 0.04) *p* = 0.5089	−0.15 (− 0.232, − 0.070) ***p*** **= 0.0003**
	3 d	−0.40 (− 0.47, − 0.33) ***p* < 0.0001**	0.00 (− 0.07, 0.08) *p* = 0.9298	−0.40 (− 0.505, − 0.300) ***p* < 0.0001**
Study 3	BL	2.44 (0.046)	2.49 (0.049)	
	0 d	−0.69 (− 0.80, − 0.57) ***p* < 0.0001**	−0.09 (− 0.20, 0.03) *p* = 0.1281	−0.60 (− 0.759, − 0.435) ***p* < 0.0001**
	3 d	−1.44 (−1.57, −1.31) ***p* < 0.0001**	−0.13 (− 0.26, 0.01) *p* = 0.0630	−1.31 (− 1.500, − 1.128) ***p* < 0.0001**
Tactile threshold				
Study 1	BL	10.00 (0.000)	10.00 (0.000)	
	0 d	+4.00 (2.68, 5.31) ***p* < 0.0001**	+ 3.60 (2.29, 4.92) ***p*** **< 0.0001**	0.39 (−1.465, 2.253) *p* = 0.6770
	3 d	+ 15.22 (12.83, 17.61) ***p* < 0.0001**	+ 3.92 (1.55, 6.29) ***p*** **= 0.0013**	11.30 (7.937, 14.662) ***p* < 0.0001**
Study 2	BL	12.66 (0.399)	13.42 (0.405)	
	0 d	+ 1.67 (1.14, 2.19) ***p* < 0.0001**	+ 0.23 (−0.30, 0.75) *p* = 0.4001	1.44 (0.694, 2.188) ***p*** **= 0.0002**
	3 d	+ 3.68 (2.94, 4.41) ***p* < 0.0001**	+ 0.11 (−0.63, 0.84) *p* = 0.7775	3.57 (2.531, 4.614) ***p* < 0.0001**
Study 3	BL	13.40 (0.431)	13.58 (0.453)	
	0 d	+ 9.05 (7.25, 10.85) ***p* < 0.0001**	+ 1.07 (−0.75, 2.89) *p* = 0.2461	7.98 (5.423, 10.539) ***p* < 0.0001**
	3 d	+ 25.87 (22.92, 28.82) ***p* < 0.0001**	+ 1.32 (−1.66, 4.30) *p* = 0.3816	24.54 (20.349, 28.736) ***p* < 0.0001**

Baseline values are raw means (standard error), and only include participants with a corresponding post-baseline assessment, for post-baseline visit output is obtained from ANCOVA model

*Change from baseline (95% CI) p-value

**Difference (95% CI) p-value: first-named minus second-named group such that, for Schiff sensitivity score, a negative difference favours the first-named group or, for tactile threshold, a positive difference favours the first-named group

BL: Baseline; d: day

*P*-values in bold are below the *p*=0.05 threshold

In all studies, these changes from baseline were significantly greater after 3 d for the Test product than the Control. Test versus Control differences ranged across the three studies from 0.40 to 1.31 units in Schiff sensitivity score, and from 3.57 g to 24.54 g in tactile threshold (Table 3). Furthermore, in Studies 2 and 3, the reduction in DH was significantly greater for the Test product than for the Control after a single use. The differences were greatest in Study 3; all favoured the Test dentifrice.

### Safety

In Study 1 there were three reported TEAEs, by one participant in the Test group (oral mucosal exfoliation) and two in the Control group (oral leucoplakia and lip ulceration). Of these, only oral mucosal exfoliation was considered treatment-related. There were no TEAEs in Study 2. In Study 3 there was one TEAE in the Test group (headache), that was not considered treatment-related. In all studies, all TEAEs were mild and had resolved by study end. There were no withdrawals in any of the studies due to a TEAE. No serious adverse events or incidents were reported.

## Discussion

Previous short-term studies have found advantages in DH relief when a toothpaste is applied in a focused manner to the affected sensitive teeth, a routine easily incorporated into normal toothbrushing. However, for SnF_2_, not all clinical studies have shown DH relief after just a single application by focused brushing [29]. In this report, a series of three studies showed that an experimental toothpaste containing 0.454% SnF_2_/5% STP applied by focused brushing significantly improved DH using two separate measures, after a single application (in two of the studies) and 3 d use, compared to conventional brushing with a regular toothpaste. The primary endpoint, of a difference in Schiff sensitivity score between the Test toothpaste/brushing regimen and the Control after 3 d use, was met in all studies. Across the three trials, the range in mean change in Schiff score (0.40–1.31 Schiff units) indicated there was a clinically meaningful difference between the effects of the Test and Control product toothpaste/brushing regimens [45]. The differences observed using the tactile stimulus paralleled these findings. These results compare well with previous studies employing this technique for stannous fluoride dentifrices, where effect sizes up to about 1.2 Schiff units have been reported after 3 days use [21–23].

Overall, a reduction in DH due to the Test toothpaste/brushing regimen was detected in the three studies reported here; however, differences in degree of effect were observed (Figs. 1 and 2/Table 3). This may be explained by the fact that any single study will only estimate the true difference between treatments; normal biological variation will mean some studies will over-estimate the true difference and some under-estimate. In this set of studies, after a single application, a statistically significant between-treatment difference was shown in Studies 2 and 3 for both sensitivity measures; in Study 1, however, there were no significant between-treatment changes on either measure. Only in this latter study did the Control toothpaste/brushing regimen show significant changes from baseline with both Schiff and tactile stimuli after a single application: it has been noted that both placebo and Hawthorne effects can affect DH studies and these may have occurred here [46–48]. It is also a possibility that there were more cases of DH severity reducing without treatment during Study 1 than in the other studies, suggested by the high proportion of Schiff score 3 at Baseline [46–48].

A ‘focused brushing’ technique was used by participants in the Test treatment group; those in the Control group brushed without specifically treating sensitive areas. This approach was taken to follow that of He et al. [21–23], aiming to mimic a real-world situation in which people with DH using either product would follow the brushing instructions for that toothpaste. Study 3 standardised the application time of the Test treatment during the focused brushing element of the brushing routine to 30 s, prior to whole mouth brushing. This may have contributed to the greater difference between Test and Control treatments observed, but many other factors including participant population, and time of year, also differed.

Despite the small differences observed between the studies, their results provide further evidence that SnF_2_ delivered by focused and whole mouth brushing with a toothpaste can be an effective tubule occluding agent, in line with in vitro studies [33, 37, 40]. The mechanism of action of the optimised polymer system of this formulation is postulated to be to promote stannous ion deposition and retention (as various tin salts). In vitro hydraulic conductance studies found that application of the Test toothpaste lowered fluid flow rate after both a single treatment and after three treatments over 48 h, as well as following a challenge with an acidic drink; these were statistically significantly greater than a similar commercial formulation containing SnF_2_ (unpublished findings). Moreover, fluid flow rate decreased with each subsequent treatment, suggesting cumulative effects of daily application. This is consistent with the observations in the three clinical studies presented here, in which beneficial changes in Schiff sensitivity score and tactile threshold recorded after single use were observed to increase after 3 d of consecutive use.

## Conclusions

The present study set shows that an experimental anhydrous toothpaste formulation, containing 0.454% SnF_2_ with an optimised polymer system, can reduce DH when applied using the focused brushing technique, relative to conventional brushing with a regular fluoride toothpaste, after only a single use. This reduction in DH builds with subsequent twice-daily applications over the ensuing 3 d. The short-term, cumulative nature of this benefit should meaningfully improve oral health-related quality of life for people with dentine hypersensitivity.

## Abbreviations

AE: Adverse event
ANCOVA: Analysis of covariance
BL: Baseline
CI: Confidence intervals
DH: Dentine hypersensitivity
EAR: Erosion, abrasion and/or gingival recession
ITT: Intent-to-treat
MGI: Modified Gingival Index
OST: Oral soft tissue
PP: Per-protocol
SD: Standard deviation
SMFP: Sodium monofluorophosphate
Sn[II]: Tin [II]
Sn[IV]: Tin [IV]
SnF_2_: Stannous fluoride
STP: Sodium tripolyphosphate
